# Establishment of Shoot Cultures of *Nepeta curviflora* Boiss., Scale-Up in a Nutrient Sprinkle Bioreactor and Phytochemical Analysis

**DOI:** 10.3390/ijms262311409

**Published:** 2025-11-25

**Authors:** Ewelina Piątczak, Klaudia Okońska, Joanna Kolniak-Ostek, Grażyna Szymańska, Ewa Kochan

**Affiliations:** 1 Department of Pharmaceutical Biotechnology, Faculty of Pharmacy, Medical University of Lodz, Muszyńskiego 1, 90-151 Lodz, Poland; grazyna.szymanska@umed.lodz.pl (G.S.); ewa.kochan@umed.lodz.pl (E.K.); 2 Department of Pharmaceutical Biotechnology (Student Scientific Association), Faculty of Pharmacy, Medical University of Lodz, Muszyńskiego 1, 90-151 Lodz, Poland; klaudia.okonska@gmail.com; 3 MSD Poland, Chlodna 51, 00-867 Warsaw, Poland; 4 Department of Fruit, Vegetable and Plant Nutraceutical Technology, Wrocław University of Environmental and Life Sciences, Chełmońskiego 37, 51-630 Wroclaw, Poland; joanna.kolniak-ostek@upwr.edu.pl

**Keywords:** cytokinins, hyperhydricity, *Nepeta curviflora*, nutrient sprinkle bioreactor, phenolic compounds, shoot culture

## Abstract

Shoot cultures of *Nepeta curviflora* were used to test the effect of 6-benzylaminopurine (BAP) and riboside 6-benzylaminopurine (r-BAP) on the growth and production of phenolic compounds. The best multiplication was achieved on agar Murashige and Skoog medium (MS) with r-BAP (1.5 mg/L), where 74% of explants produced about eight axillary shoots. The fresh weight (FW) was about 0.6 g/tube (24 g/L) and the dry weight (DW) was about 0.04 g/tube (2 g/L). To scale up the culture, the shoot culture was grown for the first time in a nutrient sprinkle bioreactor. After 4 weeks of culture, the multiplication rate (8.13) was higher than that observed in glass tubes (8.03). The fresh biomass was 113.2 g/bioreactor (75.5 g/L) and the dry mass was 14 g/bioreactor (9.3 g/L). Extracts from obtained plant material were analyzed by the UPLC/DAD/qTOF-MS technique. A total of 32 phenolic compounds were identified and quantified. The total content of compounds ranged from 600 to 1400 mg/100 g of dry weight (DW), depending on the culture conditions. In the profile of shoot cultures, rosmarinic acid was dominant, whereas prolithospermic acid was mostly noted in extract from aerial parts of the plant obtained from the field.

## 1. Introduction

*Nepeta curviflora* Boiss. (syn. *Glechoma curviflora* Boiss.) is a perennial species that naturally occurs in the Middle East. It is known in traditional medicine for its benefits in the treatment of nervous disorders, high blood pressure, rheumatic pain, fever, and upper respiratory system. Additionally, antioxidant, phytotoxic and antihelmintic activities have also been reported [1,2,3,4]. Extracts obtained from this plant were found to be rich in phenolic compounds such as rosmarinic acid, caffeic acid, apigenin, and terpenes such as betulin, betulinic acid, ursolic acid, oleanolic acid as well as nepetalactone—an iridoid characteristic of the genus *Nepeta* [4,5]. Sesquiterpenes and monoterpenes were isolated from the essential oil as the main components and include compounds such as β-caryophyllene, γ-murolene, β-farnesene and nepetalactones. Additionally, essential oil obtained from this species also contains valuable metabolites, including aliphatic hydrocarbons, fatty acids, esters, and spironoalkanes [2,3,4,6]. Such a rich composition of secondary metabolites induces the biological activity of *N*. *curviflora*, including antioxidant, lipase inhibition, α-amylase inhibition, α- glucosidase inhibition activities as well as anti-proliferative and anti-migratory properties against cervical cancer cells [6].

Organ cultures, especially shoots, are one of efficient methods of obtaining large biomass of valuable plant material. In vitro conditions allow us to conduct breeding completely independently of weather conditions or seasons and to obtain valuable secondary metabolites, often within a shorter period of time and in larger quantities than from field cultivation. Isolation from pathogens, which are a real obstacle in standard breeding, is also very important. Additionally, plants grow faster in constant, repeatable conditions, which may allow for unlimited access to plant material. This is crucial for rare and endangered endemic species that have special breeding requirements and despite promising composition of secondary metabolites, their field cultivation is not possible in other climatic zones [7,8].

However, the requirement to use semi-solid media, a large number of culture vessels and periodic transfer of plant material to fresh media after several weeks make conventional micropropagation very laborious and expensive [9]. Therefore, scaled-up and automated methods are preferred to increase multiplication rates and minimize production costs [10]. Cultivation in bioreactors, which allows for obtaining medicinal plants and its metabolites for commercial purposes, may be an alternative to conventional cultivation [9].

Many different types of bioreactors have been developed so far, but one of the most promising for plant in vitro cultures is a nutrient sprinkle bioreactor. In this type of bioreactor, the plant material is placed on a mesh and is periodically sprayed with the medium by using a pump. This solution provides good access to both oxygen and nutrients. In addition, spraying the medium through a nozzle eliminates occurrence of shear forces, which allows for uninhibited cultivation of delicate plant tissues [11]. Several shoot cultures of other plant species have been cultivated in this type of bioreactor, for example, *Salvia officinalis* [12], *Rehmannia glutinosa* [13], *Schisandra chinensis* [14], and *Dracocephalum forrestii* [15].

In the present study we evaluate the morphogenic responses of shoot tip explants of *N. curviflora* to two selected cytokinins, 6-benzylaminopurine (BAP) and 6-benzylaminopurine riboside (r-BAP), in various concentrations to obtain shoot cultures of the endemic plant species. The two cytokinins were chosen because of their high biological activities in shoot initiation and multiplication as well as high chemical stability. An auxin–IAA was also used in the experiments because of its synergistic coordination of plant morphogenesis. An interaction between BAP and r-BAP modulates the expression of key regulatory genes, enhances nutrient uptake, and maintains optimal cellular metabolism, ultimately improving regeneration efficiency and morphogenetic stability. Moreover, auxins can induce cytokinin biosynthesis genes, and cytokinins can modulate auxin transport [16,17]. The main aim of the study was a successful attempt of increasing the scale of the shoot culture in a 10 L nutrient sprinkle bioreactor. Additionally, methanolic–aqueous extracts of the plant material derived from different cytokinin treatment, as well as from shoots derived from the bioreactor were analyzed using the UPLC-DAD/ESI-MS/MS technique. For comparison purposes, methanolic–aqueous extract from aerial parts of field-grown plants was also investigated. This report is the first one describing in vitro cultures of *N. curviflora*, established both in tubes and in the bioreactor, as well as a phytochemical analysis of extracts obtained from plant material from in vitro cultures.

## 2. Results and Discussion

### 2.1. Effect of Cytokinins on Shoot Multiplication

**Figure 1:**
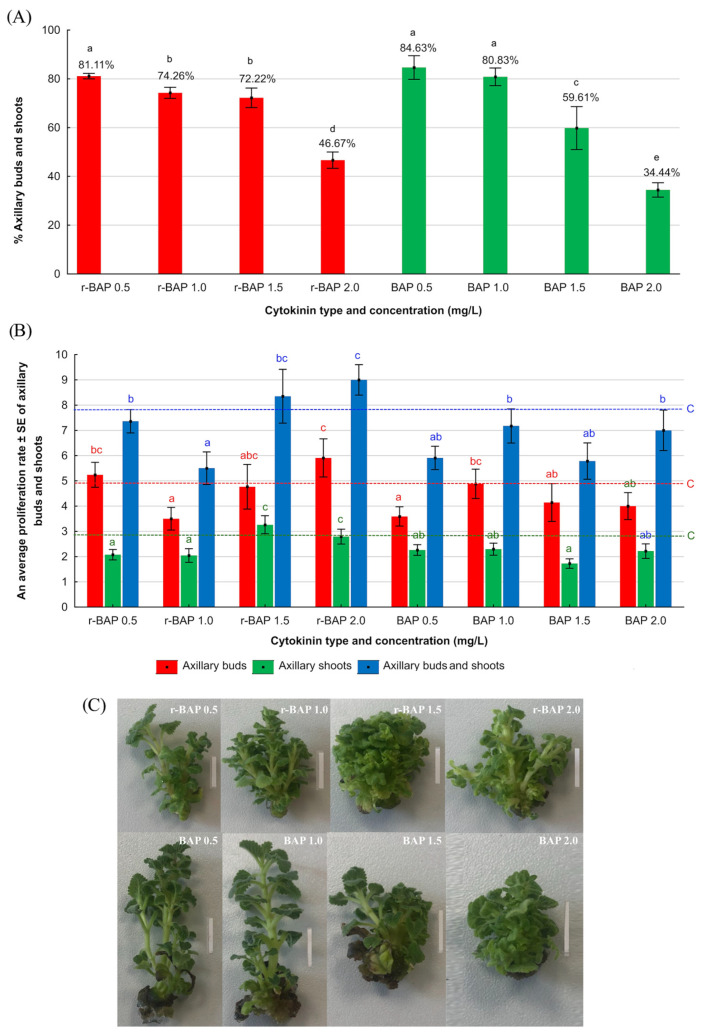
Effect of cytokinin type and concentration on (**A**) the percentage of proliferation response, (**B**) the proliferation rate, and (**C**) morphology of *N. curviflora* shoots after 4 weeks of culture on agar MS medium supplemented with IAA (0.1 mg/L) and r-BAP or BAP in concentration 0.5; 1.0; 1.5 or 2.0 mg/L. Bars = 1 cm. Bars with the same letters within a series indicate no significant differences at *p* ≤ 0.05 in the Kruskal–Wallis test. c—control conditions (MS medium with 0.1 mg/L of IAA (without cytokinin)). Data are means of six subcultures (10 single samples per single subculture per each medium variant).

**Figure 2:**
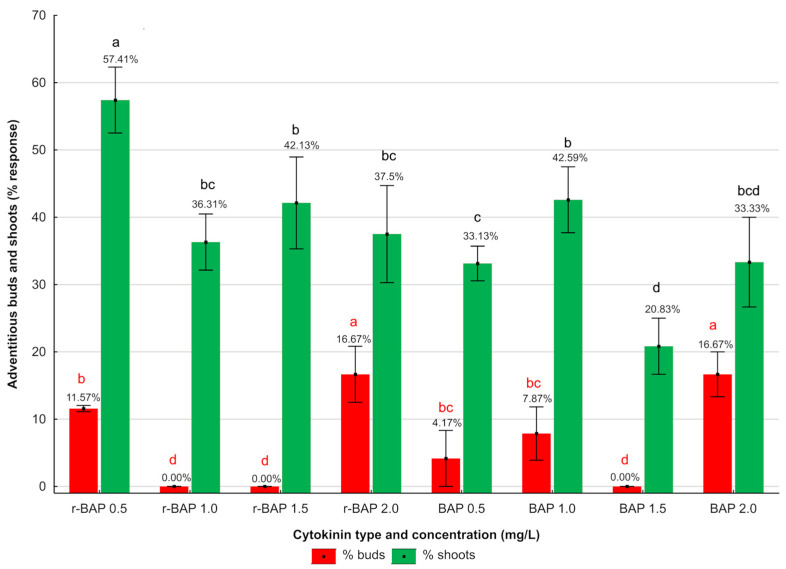
Effect of cytokinin type and concentration on the percentage formation of adventitious buds and shoots of *N. curviflora* cultivated for 4 weeks on MS medium with IAA (0.1 mg/L) and r-BAP or BAP. Bars with the same letters within a series indicate no significant differences at *p* ≤ 0.05 in the Kruskal–Wallis test.

The cytokinins used for shoot multiplication (BAP or r-BAP) of *N. curviflora* were selected on the base of the literature data and turned out to be the most effective for shoot multiplication of other species from the Lamiaceae family, including *Dracocephalum forrestii* [18], *Scutellaria altissima* [19], *Salvia officinalis* [20], *Salvia bulleyana* [21], and *Salvia viridis* [22]. The effects of cytokinins on shoot proliferation are presented in Figure 1 and Figure 2. It was observed that after 4 weeks of culture, both the type and concentration of cytokinin exhibited an effect on the multiplication and morphology of *N. curviflora* shoots in in vitro culture. However, the effect of concentration was more pronounced than the effect of the type of used cytokinin. The results were compared with the control (explants cultured on MS medium with IAA (0.1 mg/L) without cytokinin).

It was observed that the percentage of explants responded by generating new buds and/or shoots decreased when the cytokinin concentration in MS medium increased. The value ranged between 34 and 47% for the highest cytokinin concentrations (2 mg/L) and between 81 and 85% for the lowest cytokinin concentrations, regardless of its type. Explants from most media variants (except those with a cytokinin concentration of 2 mg/L) were characterized by a higher percentage of responses compared to the control sample (57.69%) (Figure 1A).

As shown in Figure 1B, after 4 weeks of culture, the highest proliferation rate, identified as a sum of shoots and buds (8.70), was observed on shoots growing in the presence of 2 mg/L of r-BAP. However, only 46.67% of explants growing on this medium produced lateral shoots and buds, which makes this medium not really effective for multiplication of plant species. The explant growing on medium with lower r-BAP concentration (1.5 mg/L), gave on average 3.26 new lateral shoots and on average 4.76 lateral buds, which results in the total multiplication rate of 8.03. In addition, proliferative response of this variant was observed in 72% of the explants (Figure 1A). Moreover, a statistical analysis did not show any significant differences (at *p* ≤ 0.05) between the two mentioned r-BAP concentrations in the number of multiplied shoots and buds. Therefore, 1.5 mg/L of r-BAP was selected as the most effective concentration for *N. curviflora* shoot multiplication. BAP as a cytokinin was not as effective as r-BAP in multiplication of *N. curviflora* shoots (Figure 1). With regard to media with BAP, the total number of axillary shoots and buds were not higher than 7.5 (Figure 1B). This advantage of r-BAP over BAP may be attributed to a modification of the r-BAP molecule, consisting of an additional substituent (ribose), present at the N9 position of the BAP ring, which according to some authors [23,24,25], may increase the effectiveness of this cytokinin contributed by increased resistance to degradation by N9-glucosylation. Such resistance effectively slows down the metabolism of r-BAP and has a more effective and longer impact on the plant. r-BAP and other ribosilated cytokinins act as transportable cytokinin forms in plant tissues. In in vitro cultured shoots, they are generally more stable than free bases due to lower susceptibility to cytokinin oxidase/dehydrogenase degradation [24,25]. Once taken up—likely via purine or nucleoside transporters—they can be enzymatically converted to active free bases, influencing morphogenesis and shoot proliferation [26,27]. Thus, r-BAP offers improved bioavailability, the balance between uptake, conversion, and degradation, which determines effective, more consistent, and reproducible growth response in tissue culture systems. Although the highest achieved multiplication rate in *N. curviflora* shoots (8.07) was not so high, and it could be a kind of limitation, the results were quite effective among the genus *Nepeta.* Previously, an almost four times lower multiplication rate (ca. 2.5) was obtained for another representative of the catnip genus, *N. asterotricha* [28] on media enriched with BAP at concentrations: 0.5, 1.0, and 2.0 mg/L. However, the authors do not clearly state whether they included only shoots or both shoots and lateral buds in the proliferation rate. *N. nuda* is another species from the same genus for which the multiplication rate appeared to be lower than the one observed in this study and amounted to 6 on a medium enriched with 6-(benzylamino)-9-(2-tetrahydropyranyl)-9H-purine—BPA (0.8 mg/L) [29].

An increased number of deformed shoots that was observed along with an increase in the cytokinin concentration in the medium, up to 67% for the highest concentration, which was applied, may be another limitation. There are literature reports about the negative impact of increasing the concentration of cytokinins, mainly BAP, on shoot morphology, e.g., the appearance of hyperhydricity [19]. Most of studies reported a disadvantageous impact of shoot hyperhydricity on plant morphology and physiology [30,31]. Many strategies are involved in reduction in the phenomenon in shoot cultures, for example, improvement of ventilation, increase in agar concentration, modification of plant growth regulator type and concentration, and increased medium osmolarity [30]. However, there are also reports where hyperhydric shoots contributed to higher levels of some secondary metabolites, including phenolic compounds, than shoots with normal morphology [32,33]. Therefore, in case of *N. curviflora*, further studies are necessary to investigate the effect of hyperhydricity on the metabolite profile in the species. In case of the reduction of metabolite production, the best method for reducing the phenomenon from the shoot culture should be developed.

In addition to the process of the formation of axillary buds and shoots, some explants showed a formation of callus (mainly at the base of the main shoot), from which adventitious buds and shoots were formed through indirect organogenesis (Figure 2). Not every explant responded by producing adventitious buds and shoots. The highest percentage of adventitious shoots (57.41%) was produced by explants growing on medium enriched with r-BAP (0.5 mg/L), with the lowest (20.83%) on medium with BAP (1.5 mg/L). The other variants, regardless of cytokinin concentration, had a similar effect on the production of adventitious shoots. No adventitious buds were observed on explants growing on media containing r-BAP (at concentrations of 1.0 or 1.5 mg/L) and BAP at a concentration of 1.5 mg/L. The highest percentage of responses (16.67%) was observed on explants growing on medium enriched with r-BAP or BAP (2.0 mg/L) (Figure 2). An average number of adventitious buds and shoots ranged from 1.6 to 2.3, but there were no significant differences between the type and concentration of cytokinin. Explants cultured under control conditions (MS medium only with and auxin) did not form any adventitious buds or shoots.

### 2.2. Fresh (FW) and Dry Weight (DW) Estimations

**Figure 3:**
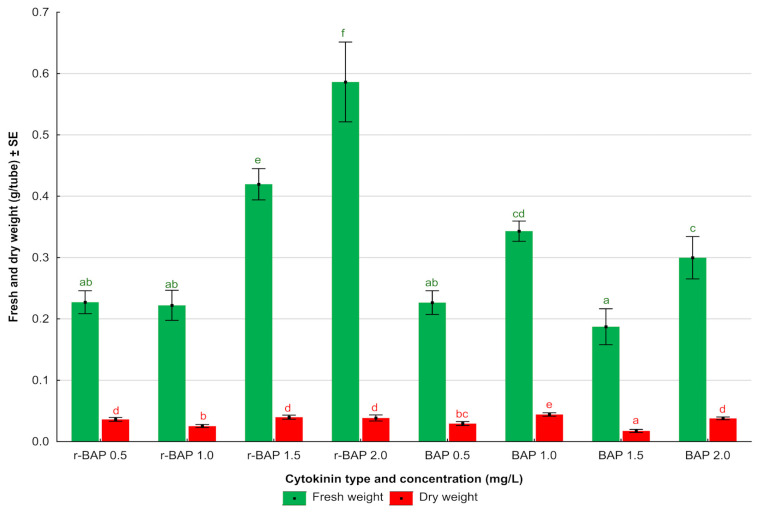
Effect of cytokinin type and concentration on average fresh and dry weights (g/tube) of *N. curviflora* cultivated for 4 weeks on MS medium with IAA (0.1 mg/L) and r-BAP or BAP. Bars bearing the same letters within each series indicate no significant differences at *p* ≤ 0.05 in Kruskal–Wallis test. Data are means of six subcultures (10 single samples per single subculture per each medium variant).

After 4 weeks of culture, fresh and dry weights (FW and DW, respectively) in g/tube were estimated (Figure 3). It was observed that shoots growing on media enriched with r-BAP were characterized by higher average values of dry and fresh weight (from 0.22 to 0.58 g/tube FW and from 0.025 to 0.040 g/tube DW) compared to shoots growing on media with BAP, where the average FW ranged from 0.19 to 0.34 g/tube and DW ranged from 0.017 to 0.044 g/tube. The highest value of an average FW was noted for shoots growing on r-BAP (2.0 mg/L). There are literature reports on cultures where fresh biomass yields were both higher and lower than in the cultures described in the paper. For example, shoot cultures of *D. forrestii* [18] characterized with higher FW yield (0.93–3.45 g/tube) can be quoted here. On the other hand, a lower yield of FW (0.2–0.3 g/tube) than that observed in the present study was earlier achieved in the shoot culture of *S. viridis* [22]. With regard to DW in cultures of *N. curviflora*, the highest value was noted for shoots growing on r-BAP (1.5 mgL), but the result was not statistically different from most other variants. The lowest values of dry and fresh biomass were noted for shoots growing on BAP (1.5 mg/L) (Figure 3). Previously, shoots of *S. viridis* [22] cultured on media with BAP and r-BAP gave comparable results in terms of DW than those we achieved for *N. curviflora.* On the other hand, shoots of *S. bulleyana* [21] gave significantly higher DW yields (up to 0.1 g/tube) than those presented in this paper for *N. curviflora*.

In our study, based on the frequency of proliferative response, multiplication rate, and dry and fresh weight, we chose MS medium with 1.5 mg/L r-BAP and 0.1 mg/L IAA to scale up the culture and further shoot multiplication in a nutrient sprinkle bioreactor (10 L volume).

### 2.3. Shoot Multiplication in a Nutrient Sprinkle Bioreactor

**Figure 4:**
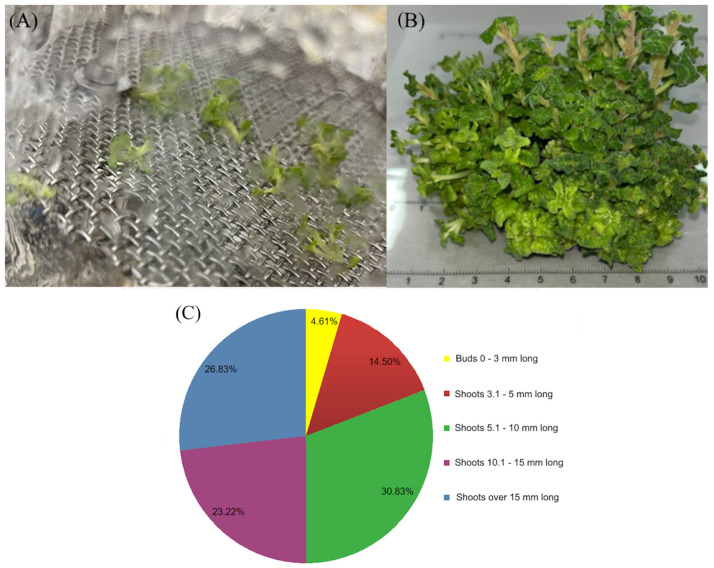
*N. curviflora* shoots growing in the nutrient sprinkle bioreactor in liquid MS medium with IAA (0.1 mg/mL) and r-BAP (1.5 mg/mL): (**A**)—inoculum; (**B**)—biomass after 4 weeks of growth; (**C**)—average percentage distribution of shoot lengths after 4-week growth.

After 4 weeks of cultivation, an average of 8.13 shoots and buds were obtained per single explant. The morphology was presented in Figure 4A,B. The obtained result is slightly higher than the multiplication rate for shoots in tubes (average 8.03) on the same medium (Table 1). This value is rather low compared to that of other species grown in the same type of bioreactor, e.g., *Rehmannia glutinosa* [13], where the multiplication rate was 21 and *D. forrestii* [34], where the multiplication rate was 69.7. On the other hand, *S. officinalis* [12] shoots multiplied worse than *N. curviflora*, reaching a multiplication rate of 4.4. The reasons for the more efficient multiplication of *N. curviflora* shoots in the nutrient sprinkle bioreactor than in glass tubes are probably better aeration of the culture and better availability of nutrients from the medium sprayed with a nozzle than from the solid medium [21,23]. The number of shoots exhibiting hyperhydricity or deformation differed between passages and ranged from 25 to 67%. This is a fairly common problem in liquid medium cultures, which can be caused by high relative humidity inside the culture vessel and hydration of the culture, poor gas exchange, poor cell-wall lignification, and increasing uptake of water as an effect of high cytokinin concentrations, insufficient light, or even genotype sensitivity. Symptoms of hyperhydration include chlorophyll deficiency, the accumulation of large starch granules in plastids, and excessive cellular hydration caused by excess fluid in the intercellular spaces. Additionally, decreased cell adhesion, the formation of larger intercellular spaces in the mesophyll, hypolignification, and reduced formation of the epicuticular layer on the leaf surface are observed. This is accompanied by changes in enzyme activity and protein synthesis resulting from disruption of normal metabolic processes. Compared to healthy plants, hyperhydrous plants also exhibit damage to cell membranes and their structure, reduced cell wall thickness, reduced mitochondrial counts, increased intercellular spaces, increased cell vacuolation, and abnormalities in the structure of vascular tissues [19,30,31]. For example, in *S. officinalis* culture [12], all multiplied shoots showed hyperhydricity, while in *D. forrestii* [34], this phenomenon was observed for 24% of the shoots. However, there are literature reports on shoots with normal morphology, e.g., *Rehmannia glutinosa* [13].

After 4 weeks of *N. curviflora* shoot culture in the bioreactor, the average FW was 113.2 g/bioreactor and dry biomass was 13.99 g/bioreactor, which, in relation to the inoculum means an 79.7-fold increase for FW and an 87.5-fold increase for DW, and after conversion to 1 L of medium, the value corresponds to 75.47 g/L of fresh mass and 9.32 g/L of dry mass. The weight is 3.5 times and 4.7 times higher, respectively, than the weight of fresh and dry biomass of shoots obtained in tubes (after conversion to 1 L of medium) (Table 1, Figure 4).

The growth index for the dry mass was on average 86.92, which is higher than the values observed for *S. officinalis* [12], where the growth index for dry weight was 45, and *D. forrestii* [34], for which the growth index was 44.55. For the fresh weight, the growth index was on average 78.33, and the value is a higher than the one observed earlier in studies on *S. officinalis* [12], for which the growth index was 42, and *D. forrestii* [34], characterized by a growth index of 43.48. These values were higher than the growth indexes obtained for shoots grown in test tubes by 1.6 times in comparison to fresh mass and 2.9 times in comparison to dry biomass (Table 1). The shoots multiplied in the bioreactor were divided into several classes, depending on their length (Figure 4C). The biggest class (30.83%) included shoots that were 5.1–10 mm long, whereas the smallest class (4.61%) included the shortest shoots, i.e., 3.1–5 mm long. Only 26.8% of shoots were longer than 1.5 cm (Figure 4C). This indicates a significant effect of added cytokinin and inhibition of apical dominance. This phenomenon is often observed in in vitro cultures grown in the presence of cytokinin in the medium. Similar results were observed in the culture of *D. forrestii* [34], where most of the shoots were not longer than 10 mm.

### 2.4. Phytochemical Studies

Shoot cultures of *N. curviflora,* carried out in tubes and a nutrient sprinkle bioreactor, were used for the first time in this study to determine the composition of secondary metabolites. In addition, aerial parts of field-grown plant were also studied. The presence of 32 phenolic compounds were detected and identified under negative electrospray ionization conditions [M-H]- based on their retention times, molecular weights, and mass (*m*/*z*) fragmentation patterns (Table 2). The ingredients included tartaric acid derivative (peak no 1), 14 phenolic acids (peaks no 2, 7, 10–11, 15, 16, 18, 21–23, 25–28), derivative of rosmarinic acid (peak no 24), 9 phenolic glycosides (peaks no 4–6, 8–9, 12, 17, 19–20), 4 esters (peaks no 13–14, 29–30), one iridoid (peak no 3), and 2 alkaloids (peak no 31–32). All the compounds were present in field-grown plants and shoot cultures cultivated in tubes, whereas in bioreactor shoot cultures, three metabolites identified as benzoyl tartaric acid (peak no 1), ethyl caffeate (peak no 14), and clinopodic acid A isomer II (peak no 27), were absent. In addition, metabolites belonging to the flavonoid group were not detected in any plant material examined in this study. In contrast, Rabee et al. [1] identified apigenin and its derivatives in aerial parts of *N. curviflora.* Furthermore, known flavonoid compounds such as naringenin, apigenin, luteolin, and its derivatives were detected in *Nepeta baytopii* and in many other species of *Lamiacaeae*, which also often contain rutin and quercetin [35,36,37,38].

On the other hand, rosmarinic acid (RA), caffeic acid (CA), ferulic acid (FA), phaselic acid (PhA), or syringic acid (SA) have been previously identified in other species of *Nepeta,* such as *N. grandiflora*, *N. nervosa*, *N. pannonica*, *N. parnassica*, *N. sibirica*, *N. sibthorpii*, *N. spicata*, *N. ernesti-mayeri*, and *N. mussinii* [39]. RA (peak no 23) demonstrated a protonated molecular ion at *m*/*z* 359. Its fragmentation leads to a formation of three characteristic ions at 197, 161, 135 (*m*/*z*). Compound 11 (caffeic acid) presented its main ion at *m*/*z* 179 with fragment 135 (*m*/*z*) and metabolite 13 (phaselic acid) showed a parent ion at *m*/*z* 259 and fragmentation ions *m*/*z* 179 and 135. An UHPLC-MS analysis of methanolic extracts of species such as: *N. grandiflora*, *N. nervosa*, *N. nuda*, *N. pannonica*, *N. parnassica*, *N. sibirica*, *N. sibthorpii*, *N. spicata*, *N. ernesti-mayeri*, *N. mussinii*, and *N. baytopii* showed the same cleavage products of RA, CA, and PhA [38,39,40]. Moreover, the studies conducted by Sharma et al. [41] confirmed a formation of the same fragment ions as those presented in this paper for SA (syringic acid), SAB (salvianolic acid B), SgA (sagerinic acid), and MR (methyl rosmarinate). Petrova et al. [33] showed clearly separated peaks identified as clinopodic acid A –CAA- (with *m*/*z* [M-H]- at 343 and fragments *m*/*z* 161, 135, 197, and 179), caffeic acid hexoside—CAH-(with *m*/*z* [M-H]- at 341 and fragments *m*/*z* 179 and 161),—dihydroxybenzoic acid hexoside –DAH- (with *m*/*z* [M-H]- at 315 and fragments *m*/*z* 153, 109, 123), nepetoidin B2 –NB2- (with *m*/*z* [M-H]- at 313 and fragments *m*/*z* 161,133), and benzoyl tartaric acid –BTA- (with *m*/*z* [M-H]- at 253 and fragment *m*/*z* 122) in *Nepeta nuda*. These results were consistent with the fragmentation products obtained in our analysis in relation to these compounds (CAA, CAH, DAH, NB2, and BTA).

**Figure 5:**
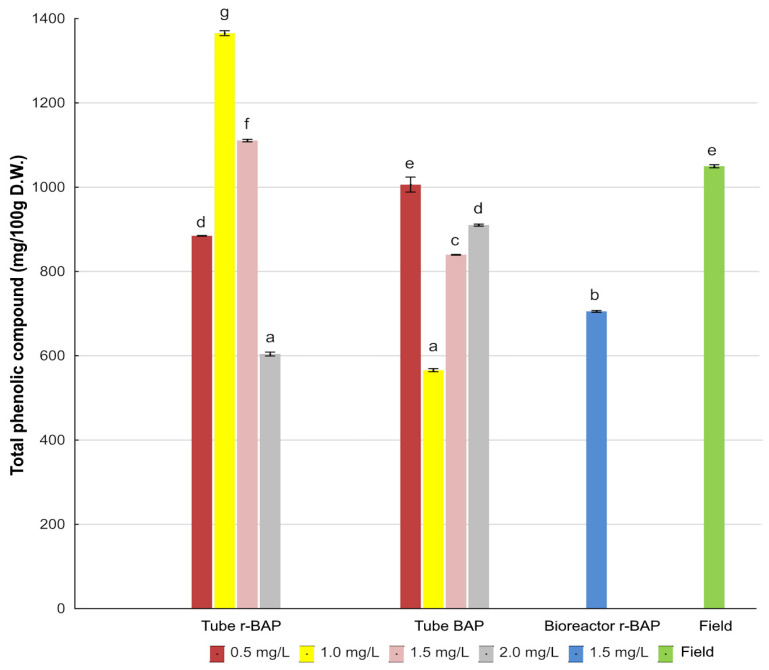
Total phenolic compounds (mg/100 g DW) in methanolic–aqueous extracts from shoot cultures (MS medium with 0.1 mg/L IAA and BAP or r-BAP) and field-grown plants of *N. curviflora.* Bars bearing the same letters within each series indicate no significant differences at *p* ≤ 0.05 in Kruskal–Wallis test.

A quantitative investigation demonstrated that *N. curviflora* shoots cultivated in tubes on medium with 1.0 mg/L r-BAP were found to be the richest source of detected phenolic compounds. Their total content was 1365.36 ± 9.3 mg/100 DW (Figure 5). This level was 1.3-fold higher than that observed in field-grown material. In contrast, cultures in vitro of *N. nuda* contained less phenolic compounds compared to the population of plant growing in situ and ex vitro (in a greenhouse) [40].

**Figure 6:**
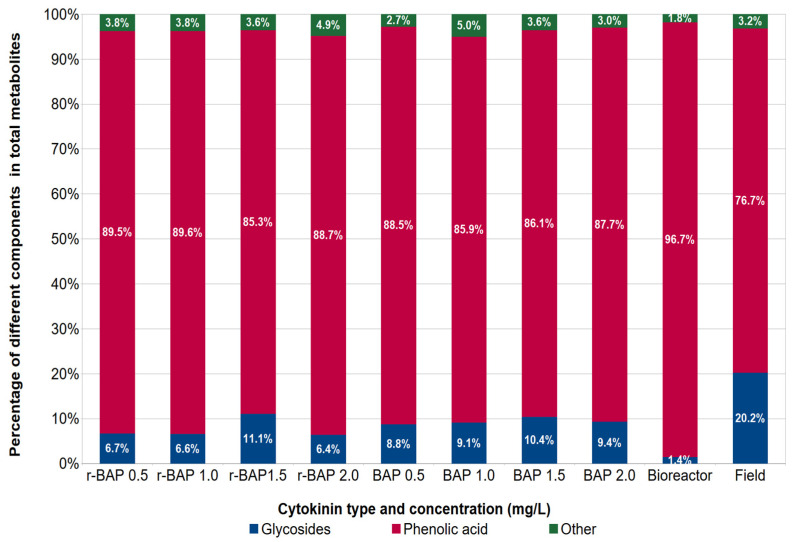
The percentage of phenolic compound groups in relation to all studied metabolites in methanolic–aqueous extracts from shoot cultures and field-grown plants of *N. curviflora*.

It was observed that phenolic acids, which quantitatively predominated, and, regardless of the sample origin, constituted from 76.7% (field plants) to 96.7% (bioreactor cultures) of the total compounds content, appeared to be the main fraction of detected metabolites (Figure 6). The percentage of the glycoside group in relation to all studied metabolites ranged between 1.4 and 20.2%. In this case, the highest percentage was found in ground plants (20.2%) and the lowest one in bioreactor samples (1.4%) (Figure 6).

**Figure 7:**
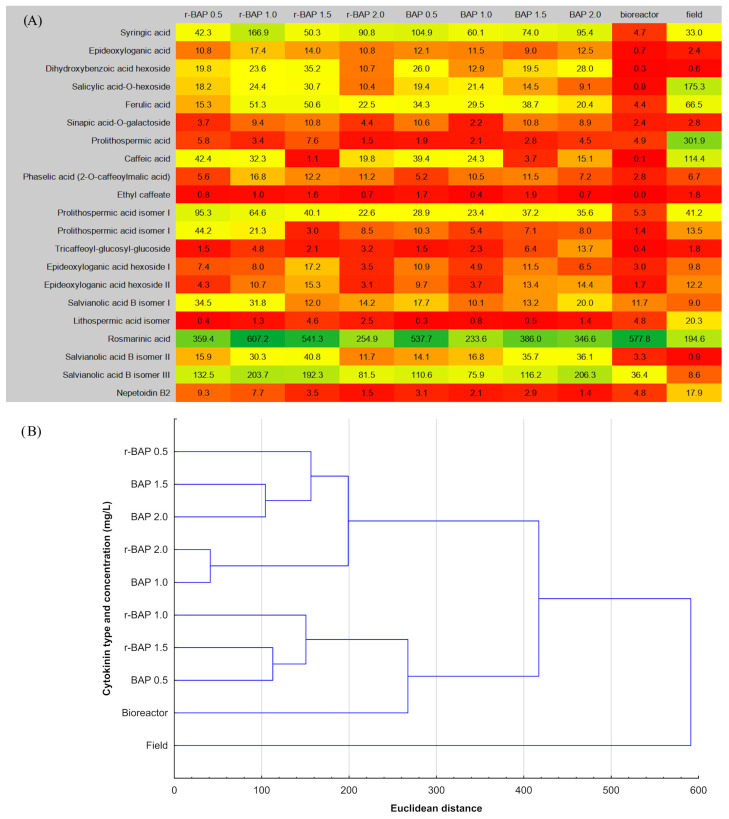
(**A**) Heat map of phenolic compounds in methanolic–aqueous extracts from shoot cultures and field-grown plants of *Nepeta curviflora*. The numbers express metabolite content (mg/100 g DW). The map includes only those compounds whose quantity in at least one of the tested samples was not lower than 10 mg/100 g DW. (**B**) Tree diagram (single linkage, Euclidean distance with standard approach) demonstrating similarities of the profile of the tested compounds under different growth conditions of *N. curviflora*.

In this study, the level of individual components was also determined. The metabolite content varied depending on the used cytokinin, its concentration, and growth conditions of plant material (Table S1, Figure S1). A comparative analysis of phytochemical profiles revealed that RA (rosmarinic acid) was a predominant ingredient in all tested samples. Its amount was 38–52.5% of all compounds in shoot cultivated in tubes, and its level was the highest in cultures growing on MS medium with r-BAP (1 mg/L). The value was as high as 607 mg/100 g DW, which was above 3 times higher than in field-grown plants (194.63 mg/100 g DW) (Table S1). On the other hand, the highest percentage content was noted in bioreactor cultures (85.3% of the total of phenolic compounds, 577.83 mg/100 g DW). The lowest level of RA was observed in field-grown plants (18.5%; 194.63 mg/100 g DW) (Figure 7A, Table S1, Figure S1). RA is a very valuable secondary metabolite exhibited several important pharmacological activities, mainly antioxidant, antiviral, antimicrobial, anti-inflammatory, anticancer, neuroprotective, and hepatoprotective [42,43,44].

Qaralleh [45] noted that RA constituted only 7.3% of all detected phenolic compounds in *N. curviflora*, which was cultivated in the Al-Karak region of Jordan and other metabolites occurred in larger quantities. The different findings, regarding the RA content in *N. curviflora* ground plants, could be associated with different conditions, such as climate, light intensity, or type of soil, in which the plants grew in Poland and Jordan. On the other hand, the levels of rosmarinic acid in leaves of ground plants can significantly differ even if the plants grew in similar condition, which was noticed in the populations of *N. nuda* originating from the Central Balkan [46]. In this case, the content of RA was lower or comparable to results obtained in this paper. Other studies, concerning in vitro cultures of three species of *Nepeta*, reported that RA was a dominant phenolic compound in *N. rtanjensis*, *N. sibirica*, and *N. nervosa* shoots [47]. However, the level of RA was significantly lower in comparison with the level observed in in vitro cultures of *N. curviflora*, described in this paper. Furthermore, compounds such as salvianolic acid B and syringic acid were also present in *N. multifida* and *N. cataria*, respectively, but similarly to our results, they were not quantitatively dominant phenolic acids [48,49,50].

The contents of nine phenolic glycosides, present in *N. curviflora* material, were demonstrated in Table S1. The findings revealed that field-grown plants of *N. curviflora* were the richest source of these components (211 mg/100 g DW expressed as the total amount of all phenolic glycosides). The most abundant metabolite was salicylic acid O-hexoside (SAH), which accounted for nearly 83% of all glycosides in traditionally cultivated plants (Figure S1). Table S1 showed levels of other phenolic compounds, detected in *N. curviflora* material. In general, these components were accumulated in small amounts, but higher contents were found in EA and nepetoidin B2. Epideoxyloganic acid is known to be present in plants belonging to the genus *Nepeta* and it has already been identified in acetone or methanolic extracts [51,52]. *N. curviflora* shoot cultures growing in tubes were a relatively rich source of this irydoid. Its amount was similar regardless of the concentration and type of the cytokinin (Table S1). Field-grown plants of *N. curviflora*, contained the highest levels of nepetoidin B2 (Figure 7A, Table S1). Nepetoidin B2 was isolated also from the aerial part of Salvia plebeia, *Elsholtzia rugulosa*, and Salvia miltiorrhiza [53,54]. Nepetoidin’s level in plants is rather low; for example, only 37 mg of this metabolite was obtained from 100 kg of *Salvia miltiorrhiza*, cultivated in the field [45]. A study by Timokhin et al. [55] showed that *N. curviflora* was an abundant source of this metabolite. Nepetoidin B exhibits several biological activities. Among them, the most interesting seems to be an anti-inflammatory effect via modulation of the NF- κB signaling pathways in macrophages [54], inhibition of LPS-stimulated nitric oxide production via modulation of iNOS pathways [56] as well as insecticidal properties against larva and adult of *Rhynchophorus ferrugineus* [57].

Our results demonstrated that quantitative profile of metabolites in extracts from in vitro cultures and field-grown material differed significantly (Figure 7A,B). A tree diagram (Figure 7B) (single linkage; Euclidean distance with standard approach) indicated that the most distinct group in terms of the quantitative composition of individual metabolites is the plant material obtained from the field (the greatest distance from the other samples). The second clearly separated sample is the bioreactor sample, although it is closer to the BAP/r-BAP clusters than the field material. The remaining samples (various concentrations of BAP and r-BAP) form a common, relatively compact cluster. Within this cluster, two subgroups can be distinguished, but there is no clear separation into independent clusters: BAP and r-BAP. The lack of a clear separation between BAP and r-BAP suggests that their influence on the biosynthesis of phenolic compounds is similar. Field material contained the most protolithospermic acid (301.9 mg/100 g DW), followed by rosmarinic acid (196.4 mg/100 g DW) and salicylic acid-O-hexoside (175.3 mg/100 g DW). Meanwhile, a clearly dominant compound in in vitro cultures was rosmarinic acid (Figure 7A). The results are not surprising. Comparative studies of plant material from conventional and biotechnological crops, conducted by other authors, also confirmed differences in the quantitative and qualitative composition of active compounds in in vitro cultures and field-grown plants [58,59,60]. In addition, our results proved that cultures grown in tubes were a richer source of different phenolic compounds in comparison to bioreactor cultures, in which the main determined metabolite was rosmarinic acid. The high rosmarinic acid content in a bioreactor, accompanied by low levels of other metabolites, can be explained by several biochemical and physiological factors. In vitro culture conditions—such as high humidity, altered gas exchange, nutrient imbalance, or mechanical stress from liquid media in bioreactors—impose abiotic stress on plant tissues. These stresses disturb cellular homeostasis, leading to the production of reactive oxygen species (ROS) and activation of defense signaling pathways (mainly via jasmonic acid, salicylic acid, and abscisic acid). Rosmarinic acid (RA) is a phenolic compound synthesized through the phenylpropanoid and tyrosine-derived pathways, both of which are part of the plant’s antioxidant and defense metabolism. Under stress, plants often redirect metabolic flux from the primary metabolism toward the secondary metabolism, especially toward phenolic antioxidants that can neutralize ROS and stabilize cellular structures. Key enzymes involved in RA biosynthesis—such as phenylalanine ammonia-lyase (PAL), tyrosine aminotransferase (TAT), and rosmarinic acid synthase (RAS)—are transcriptionally upregulated by stress-related signaling molecules (e.g., jasmonates, ROS, and ethylene). This results in elevated accumulation of phenolic intermediates and higher RA content. In bioreactor cultures, stress factors like periodic immersion, oxygen fluctuations, and shear forces can therefore act as elicitors, stimulating defense metabolism and pushing carbon flow into the RA pathway rather than into growth-related processes. This explains why, in systems such as the nutrient sprinkle bioreactor, shoots sometimes exhibit both stress symptoms (including hyperhydricity) and increased RA accumulation [61,62,63].

Some investigations [34,64,65,66] and our observations revealed that the plants from the Lamiaceae family may respond to growth conditions differently, which activates biosynthesis of different branches of phenolic compounds. The tube shoots of *N. curviflora* grew on solid medium and bioreactor organs were temporarily sprinkled with a sprayed medium. In effect, nutrient supply, aeration volume, gas phase composition, or the concentration of gaseous components (carbon dioxide, oxygen, ethylene or others) were different in both cultures. It is possible that these differences can influence the dynamics of phenolic compound biosynthesis and promote the pathway towards mainly RA (in bioreactor) or RA and other metabolites (tube cultures). For example, Döring et al. [67] revealed that, eight enzymes, among them PAL, 4-coumarate:coenzyme A ligase (4CL), TAT, and RAS, involved in RA biosynthesis, were a quick upregulation after O_3_ exposure of four-month-old cuttings of *Melissa officinalis*, which led to an increase in the RA level. Duan et al. [68] demonstrated that several genes in the anthocyanin biosynthetic pathway were upregulated by BAP cytokinin. Similarly, genes involved in cell growth and development were also upregulated by BAP, supporting the role of the cytokinin in promoting leaf growth and development and its likely regulatory role in phenolic biosynthesis [68]. We also found that the amounts of salvianolic acid B isomer III, syringic acid, prolithospermic acid, and caffeic acid, produced in test tube cultures, were slightly higher but significantly lower than the amount of RA. For better comparison of the nutrient sprinkle bioreactor system of *N. curviflora* shoots with cultures in test tubes, productivity calculations were performed and expressed as total phenolic compound per liter of medium per single day (Table S2). It was observed that the shoots cultured in bioreactor system biosynthesized 2.25 mg of phenolic compounds/L/day. The value was almost 10-fold higher than the productivity of the compounds in test tubes at the same cytokinin type and concentration. Earlier, Szopa et al. [14] achieved lower values of phenolic compound productivity (0.1 mg/L/day) after 30 days of growth and 0.08 mg/L/day after 60 days of growth of shoot cultures of *Schisandra chinensis* [14]. On the other hand, 2.5-fold higher productivity of total phenolic compounds (6.24 mg of phenolic compounds per liter of medium per single day) than achieved in the present study was achieved in transformed shoot cultures of *Dracocephalum forrestii* after 21 days of growth in a nutrient sprinkle bioreactor [34]. Such faster growth of transformed shoot culture than not transformed *N. curviflora* cultures is not surprising, because faster growth is one of the characteristic features of transformed organ cultures.

To sum up, it should be pointed out that the most favorable variant of medium for obtaining shoot biomass was not the most favorable for accumulation of phenolic compounds. Biosynthesis efficiency of these metabolites was lower for both the main compound, i.e., rosmarinic acid% and the total number of studied compounds by approximately 10% and 15%, respectively, in comparison to the medium in which the maximum amounts were determined. It must be emphasized that MS with 1.0 mg/L r-BAP enabled to produce much lower yield of dry biomass. Hence, medium containing 1.5 mg/L r-BAP should be applied in further investigations.

## 3. Materials and Methods

### 3.1. Plant Material

In vitro shoot culture was established using *N. curviflora* seeds obtained from the Jerusalem Botanical Garden. Seeds were sterilized in an aqueous solution of sodium hypochlorite 2% (*v*/*v*) for 3 min 45 s, then rinsed in sterile distilled water (3 × 10 min) and transferred to Erlenmeyer flasks containing 80 mL of sterile agar medium (0.7% *w*/*v*) according to Murashige and Skoog (MS) (Duchefa Biochemie, Haarlem, The Netherlands) [69] without the addition of growth regulators. After 4 weeks, the apical part of the shoot with the apical meristem (3–5 cm long) was cut off from the germinated seeds and transferred under sterile conditions to glass tubes containing 25 mL of MS agar medium supplemented with r-BAP (Duchefa Biochemie, Haarlem, The Netherlands) (1 mg/L) and indole-3-acetic acid—IAA (Duchefa Biochemie, Haarlem, The Netherlands) (0.1 mg/L). Every 4 weeks for 7 passages they were transplanted into fresh medium to obtain stable in vitro culture for further experiments.

### 3.2. Shoot Proliferation

*N. curviflora* shoots were multiplied by clonal propagation, using as explants 3–6 mm long tip parts with apical meristem from shoots growing in vitro. The fresh weight of the inoculum was 0.0085 g, while the dry weight was 0.0013 g. The explants were transferred individually into glass tubes (Ø 25 mm) containing 25 mL of agar (0.7% *w*/*v*) MS medium with IAA (0.1 mg/L) and one of two cytokinins (BAP or r-BAP) (Duchefa Biochemie, Haarlem, The Netherlands) at concentrations of 0.5; 1.0; 1.5; or 2.0 mg/L. Simultaneously, we cultivated shoots under control conditions, in which explants were placed individually in tubes containing MS agar medium with an addition of IAA (0.1 mg/L) as the only growth regulator. The pH of all media was adjusted to 5.6–5.8 with 0.1 M sodium hydroxide solution and 5% hydrochloric acid. The culture was kept at 25 °C under white lamps with a light intensity of 40 µM·m^−2^·s^−1^. The media were sterilized in an autoclave under the following conditions: 120 °C, 20 min, 1 atm. After 4 weeks of culture the percentage of explants giving a response in the form of multiplication of new axillary and adventitious buds or shoots, the multiplication rate, i.e., the average number of buds and shoots on single explant, the percentage of deformed shoots, and the average fresh and dry weight were determined.

### 3.3. Shoot Multiplication in the Nutrient Sprinkle Bioreactor

To scale up the culture, a 10 L nutrient sprinkle bioreactor was used to grow *N. curviflora* shoots. The explants used for shoot multiplication in the bioreactor were apical parts cut off from 4-week-old shoots, grown in glass tubes with an average fresh weight of 1.10 g and an average dry weight of 0.13 g. Liquid MS medium (1.5 L of total volume) with an addition of r-BAP (1.5 mg/L) and IAA (0.1 mg/L) was supplied to the culture with the use of a peristaltic pump (Unipan peristaltic pump type 371, Warsaw, Poland) through a nozzle placed at the bottom of the bioreactor. The medium was supplied every 1.5 h for 10 min (flow rate 112 mL/min). The average fresh weight of inoculum was 1.42 g ± 0.1 g, the average dry weight was 0.16 g ± 0.01, and the number of explants (shoot tips 0.3–0.5 mm long) was 10–20 per single bioreactor run. The study included 3 series of cultures in the bioreactor, each of which lasted 4 weeks. After 4 weeks of culture, all multiplied shoots were counted, classified by length and divided into 5 classes: 0–3 mm long, 3.1–5.0 mm long, 5.1–10 mm long, 10.1–15 mm long, 15.1 mm, and longer. The multiplication rate was calculated as a number of shoots and buds multiplied from a single explant. The morphology of multiplied shoots was also determined. In addition, biomass (fresh and dry weights—FW and DW, respectively) in g/vessel and g/L of medium, as well as the growth index both for fresh and dry weights, were also calculated.

### 3.4. Extract Preparation

Lyophilized and powdered plant material (100 mg per sample), derived from shoot cultured under all media variants (four concentrations of r-BAP and BAP), as well as shoots derived from the bioreactor as well as aerial parts of field-grown plants, were pre-extracted with chloroform (20 mL) for 12 h at room temperature. Chloroform supernatants were filtered off. Then, the dried plant material was extracted three times with a methanol–water solution at a ratio of 8:2 (*v*/*v*) according to the method described by Piątczak et al. [70]. The obtained dry extracts were stored at 4 °C until used.

### 3.5. Phytochemical Investigations

Phytochemical analyses were performed using UPLC equipped with quadrupole Time of Flight–double mass spectrometry detector (UPLC/DAD/qTOF-MS/MS) (Waters Corporation, Milford, MA, USA) as described by Zielińska et al. [71]. Polyphenolic compounds were separated by a UPLC BEH C18 column (1.7 µm, 2.1 × 100 mm, Waters Corporation, Milford, MA, USA). The mobile phase consisted of solvent A (0.1% formic acid in LC–MS grade water, *v*/*v*, Merck, Darmstadt, Germany) and solvent B (0.1% formic acid in LC–MS grade acetonitrile, Merck, Darmstadt, Germany). The retention times and spectra of the analyzed compounds were compared with those of authentic standards. The measurement of phenolic compounds was conducted using external calibration curves, employing reference compounds chosen according to the principle of structure-related target analyte/standard (chemical structure or functional group). The calibration curves were made from benzoic acid, caffeic acid, clinopodic acid A, ferulic acid, nepetoidin B, p-coumaric acid, rosmarinic acid, sagerinic acid, salvianolic acid B, sinapic acid, and syringic acid as standards at concentrations ranging from 0.05 to 5 mg/mL (precision of calibration curves not <r^2^ = 0.9998; limit of detection not <0.03 ppm).

The calibration curve for caffeic acid was used, besides its own quantification, to quantify caffeic acid hexosides, ethyl caffeate, phaselic acid, and tricaffeoyl-glucosyl-glucoside. The calibration curve for p-coumaric acid was used to quantify p-coumaric acid isoprenyl ester. The calibration curve of sinapic acid was used, to quantify sinapic acid-O-galactoside and -O-glucoside. The benzoic acid calibration curve was used, to quantify benzoyl tartaric acid and dihydroxybenzoic acid hexoside. The rosmarinic acid calibration curve was used, besides its own quantification, to quantify rosmarinic acid derivatives and methyl rosmarinate. The salvianolic acid B and clinopodic acid A calibration curves were used to quantify their own isomers. Syringic, ferulic, sagerinic acid, and nepetoidin B were quantified with their own standards. Epideoxyloganic acid derivatives were quantified as loganic acid derivatives. Lithospermic and prolithospermic acid was quantified as salvianolic acid A equivalents. The quantities of compounds were expressed as mg per 100 g of plant material dry weight (mg/100 g DW). All determinations were made in triplicate (n = 3).

### 3.6. Statistical Analysis

All data are presented as the average ± standard error (SE). The calculations were performed using Microsoft Excel 2010. An analysis of statistically significant differences was performed using STATISTICA 13.3 (TIBCO Statistica) and the Kruskal–Wallis nonparametric test for multiple groups. Data are means from six independent subcultures with 10 replicates per each subculture per each medium variant. Experiments with bioreactor were repeated three times. In the graphs, the means marked with the same letters do not differ statistically from each other for the 95% level of significance. For better visualization of phytochemical analyses, a heat map and tree map (dendrogram) were made. Data were organized in a matrix structure, where rows represent cases and columns represent variables. Then, an appropriate color scale to represent the magnitude of values was applied. In addition, hierarchical clustering was also performed based on the heat map with single linkage and Euclidean distance with standard approach, where at each step two objects with the smallest distance were combined with each other.

## 4. Conclusions

This study was the first step of investigation of *N. curviflora* shoot cultures. Given the limited occurrence of the field-grown plants and the ability to cultivate shoots under strictly controlled conditions, our research may contribute to the preservation of the species biodiversity. Our research demonstrated that the obtained cultures could become an alternative source of phenolic compounds in the future. They contained more of the studied metabolites than field-grown plants and accumulated higher levels of rosmarinic acid, known for its medicinal properties. Furthermore, conditions for scaling up shoot cultivation in bioreactors were successfully developed. Although the total content of tested metabolites in bioreactor cultures was lower than that in field-grown plants and that in mostly shoot cultures cultivated in a tube, even on the same regulator combinations, the level of the main compound, i.e., rosmarinic acid, was only 3% (588.83 mg/100 g DW) lower than its maximum amount determined in the studied plant material (607.19 mg/100 g DW). Moreover, we should bear in mind that 4.7 times higher dry biomass of shoots per one liter of culture can be obtained after 4 weeks of culture in a bioreactor rather than in glass tubes. Thus, it is possible to obtain a total of 63 mg of metabolites from 1 L of culture, whereas cultures in tubes on the same medium composition (MS medium with IAA (0.1 mg/L)) and r-BAP (1.5 mg/L) are able to generate only 7.7 mg of metabolites per one liter of culture. With regard to the other concentration of r-BAP (1 mg/L), where the total content of compounds in mg/100 g DW was the highest (almost 1400 mg/g DW), the total amounts of compounds obtained from one liter of culture is 2.6 times smaller than in a bioreactor because of low dry biomass. This phenomenon makes bioreactor cultures more effective in the overall production of phenolic compounds in shoot cultures of *N. curviflora* than those established in glass tubes. However, further research is needed, particularly improving the multiplication rate and increasing the yield of active substances using biotechnological methods such as elicitation (e.g., methyl jasmonate, salicylic acid, yeast extract) or addition of direct or indirect precursors of phenolic compound biosynthesis (tyrosine, phenylalanine) to the medium. Molecular studies should also be designed to better understand the mechanisms regulating the biosynthesis of rosmarinic acid and other phenolic compounds. The knowledge, especially at the molecular and genetic level, would enable us to increase industrial production of bioactive compounds based on the latest techniques, including metabolic engineering. Additionally expanding the metabolomic profiling to include volatile terpenoids using GC MS would give a more complete understanding of *N. curviflora* chemistry.

## Figures and Tables

**Table 1:** Growth and morphology of shoots of *Nepeta curviflora* after 4 weeks of growth in a nutrient sprinkle bioreactor in liquid MS medium supplemented with IAA (0.1 mg/L) and r-BAP (1.5 mg/L). For comparison, the results obtained in glass tubes (on the same medium) are also presented.

	Multiplication Rate ± SE	Fresh Weight (FW) (g/Vessel *) ± SE	Dry Weight (DW) (g/Vessel *) ± SE	FW (g/L) ± SE	DW (g/L) ± SE	Growth Index for FW	Growth Index for DW	Morphological Deformation and Hyperhydricity (%)
Bioreactor	8.13 ± 0.375	113.2 ± 7.09	13.99 ± 0.045	75.47 ± 4.73	9.32 ± 0.51	78.33	86.92	44.29
Glass tubes	8.03 ± 0.257	0.42 ± 0.02	0.0397 ± 0.0033	21.00 ± 1.15	1.98 ± 0.083	48.58	30.34	60.00

* vessel means bioreactor or single tube.

**Table 2:** UPLC-DAD ESI-MS detection and identification of phenolic compounds in methanolic–aqueous extracts from shoot cultures and field-grown plants of *Nepeta curviflora*.

Peak No.	Rt (min)	MS [M-H]-	MS/MS/Fragmentation Ions [M-H]-	Tentative Identification	r-BAP				BAP				Bioreactor	Field
					0.5	1	1.5	2	0.5	1	1.5	2		
1.	2.06	253.1142	122	Benzoyl tartaric acid	+	+	+	+	+	+	+	+	nd	tr
2.	2.46	197.0776	179, 135	Syringic acid	+	+	+	+	+	+	+	+	+	+
3.	2.60	359.1377	197, 153, 109	Epideoxyloganic acid	+	+	+	+	+	+	+	+	+	+
4.	2.87	315.1454	153, 109	Dihydroxybenzoic acid hexoside	+	+	+	+	+	+	+	+	+	+
5.	3.10	299.1160	137	Salicylic acid-O-hexoside	+	+	+	+	+	+	+	+	+	+
6.	3.47	341.1260	191,179,161	Caffeic acid hexoside I	+	+	+	+	+	+	+	+	+	+
7.	3.54	193.0805	149, 147	Ferulic acid	+	+	+	+	+	+	+	+	+	+
8.	3.91	341.1213	179	Caffeic acid hexoside II	+	+	+	+	+	+	+	+	+	+
9.	4.13	385.1527	223, 179	Sinapic acid-O-galactoside	+	+	+	+	+	+	+	+	+	+
10.	4.32	357.1578	315, 135	Prolithospermic acid	+	+	+	+	+	+	+	+	+	+
11.	4.42	179.0633	135	Caffeic acid	+	+	+	+	+	+	+	+	tr	+
12.	4.80	385.1551	223, 179	Sinapic acid-O-glucoside	+	+	+	+	+	+	+	+	+	+
13.	4.98	295.0790	179, 135	Phaselic acid (2-O-caffeoylmalic acid)	+	+	+	+	+	+	+	+	+	+
14.	5.10	207.0973	192, 163, 135	Ethyl caffeate	+	+	+	+	+	+	+	+	nd	+
15.	5.44	357.0998	313, 269, 207	Prolithospermic acid isomer	+	+	+	+	+	+	+	+	+	+
16.	5.65	357.0998	313, 269, 203	Prolithospermic acid isomer	+	+	+	+	+	+	+	+	+	+
17.	5.80	827.2560	664, 383, 341, 179	Tricaffeoyl-glucosyl-glucoside	+	+	+	+	+	+	+	+	+	+
18.	5.96	719.3304	359, 197, 153, 135	Sagerinic acid	+	+	+	+	+	+	+	+	+	+
19.	6.69	521.1690	503, 359, 135	Epideoxyloganic acid hexoside I	+	+	+	+	+	+	+	+	+	+
20.	6.79	521.1730	335, 289, 135	Epideoxyloganic acid hexoside II	+	+	+	+	+	+	+	+	+	+
21.	7.37	717.1961	519, 339	Salvianolic acid B isomer I	+	+	+	+	+	+	+	+	+	+
22.	7.93	537.0245	295	Lithospermic acid isomer	+	+	+	+	+	+	+	+	+	+
23.	8.09	359.1183	197, 161, 135	Rosmarinic acid	+	+	+	+	+	+	+	+	+	+
24.	8.56	567.2520	359, 161	Rosmarinic acid derivative	+	+	+	tr	tr	tr	tr	tr	+	+
25.	8.86	717.1961	519, 321, 295	Salvianolic acid B isomer II	+	+	+	+	+	+	+	+	+	+
26.	9.17	343.1200	197, 179, 161, 145, 135	Clinopodic acid A isomer I	+	+	+	+	+	+	+	+	+	+
26.	9.17	343.1200	197, 179, 161, 145, 135	Clinopodic acid A isomer I	+	+	+	+	+	+	+	+	+	+
27.	9.23	343.1183	161, 135	Clinopodic acid A isomer II	+	+	+	+	+	+	+	+	nd	+
28.	9.38	717.1961	519, 321, 295	Salvianolic acid B isomer III	+	+	+	+	+	+	+	+	+	+
29.	9.46	373.1340	197, 175, 135	Methyl rosmarinate	+	+	+	+	+	+	+	+	+	+
30.	9.80	231.1667	163, 119	p-Coumaric acid isoprenyl ester	+	+	+	+	+	tr	tr	+	tr	+
31.	10.85	313.1052	161, 133	Nepetoidin B1	+	+	+	+	+	+	+	+	+	+
32.	11.68	313.1088	161, 133	Nepetoidin B2	+	+	+	+	+	+	+	+	+	tr

Concentration of plant growth regulators in mg/L. + means that the compound is present. tr means that the compound is present in traces amounts (concentration below 0.10 mg/100 g DW). nd means that the compound was not detected.

## Data Availability

The raw data supporting the conclusions of this article will be made available by the authors on request.

## Supplementary Materials

The following supporting information can be downloaded at https://www.mdpi.com/article/10.3390/ijms262311409/s1.
