# Supplementary material for: Establishment of Shoot Cultures of Nepeta curviflora Boiss., Scale-Up in a Nutrient Sprinkle Bioreactor and Phytochemical Analysis
Source: Int J Mol Sci. 2025 Nov 25;26(23):11409. doi: 10.3390/ijms262311409 (PMC12692223; doi:10.3390/ijms262311409)
Supplement: Supplementary file 1 [file ijms-26-11409-s001.zip › Table S1.pdf]

Table S1. Content of phenolic compounds (mg/100gD.W.) in methanolic-aqueous extracts from shoot cultures and field-grown plants of *Nepeta curviflora*

		r-BAP (mg/L)				BAP (mg/L)					
**Peak no**	**Metabolite**	**0.5**	**1.0**	**1.5**	**2.0**	**0.5**	**1.0**	**1.5**	**2.0**	**bioreactor**	**field**
1	BTA	$0.63^{a} \pm 0.03$	$1.62^{b} \pm 0.11$	$1.22^{c} \pm 0.02$	$0.85^{d} \pm 0.02$	$0.98^{e} \pm 0.02$	$0.57^{a} \pm 0.04$	$0.84^{d} \pm 0.02$	$0.68^{a} \pm 0.03$	0.00	$0.05^{f} \pm 0.01$
2	SA	$42.33^{a} \pm 2.33$	$166.89^{b} \pm 1.57$	$50.31^{c} \pm 0.32$	$90.83^{d} \pm 0.43$	$104.9^{e} \pm 1.93$	$60.08^{f} \pm 0.57$	$74.05^{g} \pm 0.68$	$95.35^{h} \pm 1.70$	$4.89^{i} \pm 0.25$	$32.96^{j} \pm 0.11$
3	EA	$10.76^{a} \pm 0.08$	$17.36^{b} \pm 1.35$	$14.02^{c} \pm 0.14$	$10.78^{a} \pm 0.21$	$12.14^{d} \pm 0.11$	$11.45^{e} \pm 0.30$	$9.01^{f} \pm 0.15$	$12.46^{g} \pm 0.11$	$0.78^{h} \pm 0.22$	$2.36^{i} \pm 0.08$
4	DAH	$19.82^{a} \pm 0.18$	$23.59^{b} \pm 0.75$	$35.18^{c} \pm 0.53$	$10.67^{d} \pm 0.44$	$25.99^{e} \pm 0.46$	$12.91^{f} \pm 0.36$	$19.48^{a} \pm 0.30$	$28.03^{g} \pm 0.69$	$0.36^{h} \pm 0.06$	$0.6^{i} \pm 0.01$
5	SAH	$18.2^{a} \pm 0.15$	$24.44^{b} \pm 0.72$	$30.67^{c} \pm 0.87$	$10.39^{d} \pm 0.04$	$19.44^{e} \pm 0.20$	$21.45^{f} \pm 1.00$	$14.54^{g} \pm 0.33$	$9.07^{h} \pm 0.47$	$0.87^{i} \pm 0.33$	$175.25^{j} \pm 1.77$
6	CAH I	$3.56^{a} \pm 0.07$	$6.2^{b} \pm 0.11$	$8.69^{c} \pm 0.20$	$1.49^{d} \pm 0.13$	$5.54^{e} \pm 0.09$	$2.66^{f} \pm 0.12$	$7.59^{g} \pm 0.13$	$2.46^{h} \pm 0.11$	$0.94^{i} \pm 0.21$	$6.31^{b} \pm 0.04$
7	FA	$15.33^{a} \pm 0.14$	$51.27^{b} \pm 0.70$	$50.62^{b} \pm 0.33$	$22.45^{c} \pm 0.03$	$34.28^{d} \pm 0.19$	$29.5^{e} \pm 1.06$	$38.75^{f} \pm 0.32$	$20.44^{g} \pm 0.22$	$3.37^{h} \pm 0.51$	$66.5^{i} \pm 0.34$
8	CAH II	$0.81^{a} \pm 0.33$	$1.53^{b} \pm 0.04$	$2.08^{c} \pm 0.10$	$0.77^{a} \pm 0.07$	$2.07^{c} \pm 0.05$	$0.57^{d} \pm 0.08$	$1.62^{e} \pm 0.18$	$0.97^{f} \pm 0.04$	$0.34^{g} \pm 0.09$	$2.53^{h} \pm 0.24$
9	SAGa	$3.71^{a} \pm 0.11$	$9.39^{b} \pm 0.33$	$10.8^{c} \pm 0.12$	$4.43^{d} \pm 0.17$	$10.63^{c} \pm 0.15$	$2.23^{e} \pm 0.12$	$10.79^{c} \pm 0.41$	$8.86^{f} \pm 0.15$	$2.78^{e} \pm 0.47$	$2.83^{e} \pm 0.06$
10	PA	$5.79^{a} \pm 0.07$	$3.36^{b} \pm 0.13$	$7.55^{c} \pm 0.08$	$1.47^{d} \pm 0.06$	$1.93^{e} \pm 0.08$	$2.13^{e} \pm 0.12$	$2.76^{f} \pm 0.01$	$4.5^{g} \pm 0.10$	$6.58^{c} \pm 0.33$	$301.89^{h} \pm 4.37$
11	CA	$42.36^{a} \pm 0.77$	$32.27^{b} \pm 0.13$	$1.1^{c} \pm 0.00$	$19.77^{d} \pm 0.40$	$39.41^{e} \pm 0.59$	$24.31^{f} \pm 3.65$	$3.73^{g} \pm 0.05$	$15.11^{h} \pm 0.89$	$0.06^{i} \pm 0.03$	$114.35^{j} \pm 0.49$
12	SAGu	$0.24^{a} \pm 0.01$	$1.8^{b} \pm 0.30$	$1.12^{c} \pm 0.01$	$1.15^{c} \pm 0.04$	$3.99^{d} \pm 0.03$	$0.78^{e} \pm 0.06$	$1.93^{b} \pm 0.07$	$0.98^{f} \pm 0.11$	$0.29^{g} \pm 0.07$	$0.62^{h} \pm 0.03$
13	PhA	$5.57^{a} \pm 0.18$	$16.77^{b} \pm 0.54$	$12.19^{c} \pm 0.57$	$11.21^{d} \pm 0.1$	$5.25^{a} \pm 0.10$	$10.53^{e} \pm 0.06$	$11.47^{d} \pm 0.22$	$7.19^{f} \pm 0.03$	$2.49^{g} \pm 0.46$	$6.74^{f} \pm 0.09$
14	EC	$0.85^{ab} \pm 0.02$	$0.1^{b} \pm 0.05$	$1.56^{c} \pm 0.05$	$0.71^{a} \pm 0.07$	$1.73^{d} \pm 0.09$	$0.44^{e} \pm 0.03$	$1.93^{f} \pm 0.02$	$0.7^{a} \pm 0.01$	0.00	$1.75^{d} \pm 0.02$
15	PA I	$95.31^{a} \pm 0.19$	$64.56^{b} \pm 2.66$	$40.09^{c} \pm 0.17$	$22.61^{d} \pm 1.72$	$28.92^{e} \pm 0.36$	$23.39^{d} \pm 0.65$	$37.21^{f} \pm 0.57$	$35.55^{f} \pm 0.06$	$4.83^{g} \pm 0.27$	$41.22^{c} \pm 1.21$
16	PA II	$44.21^{a} \pm 0.89$	$21.31^{b} \pm 0.54$	$3.01^{c} \pm 0.03$	$8.53^{d} \pm 0.25$	$10.33^{e} \pm 0.07$	$5.42^{f} \pm 0.17$	$7.07^{g} \pm 0.06$	$8.02^{d} \pm 0.03$	$1.6^{h} \pm 0.17$	$13.46^{i} \pm 0.34$
17	TGG	$1.49^{a} \pm 0.11$	$4.79^{b} \pm 0.07$	$2.09^{c} \pm 0.08$	$3.21^{d} \pm 0.92$	$1.54^{a} \pm 0.12$	$2.34^{c} \pm 0.03$	$6.41^{e} \pm 0.10$	$13.72^{f} \pm 0.10$	$0.38^{g} \pm 0.04$	$1.77^{h} \pm 0.02$
18	SgA	$0.63^{a} \pm 0.04$	$1.58^{b} \pm 0.07$	$0.63^{a} \pm 0.03$	$0.7^{c} \pm 0.01$	$1.47^{b} \pm 0.04$	$0.78^{c} \pm 0.61$	$0.41^{d} \pm 0.05$	$0.47^{d} \pm 0.05$	$0.12^{e} \pm 0.05$	$0.15^{e} \pm 0.02$

19	EAH I	7.39^{a} ± 0.11	8.02^{b} ± 0.31	17.22^{c} ± 0.52	3.53^{d} ± 0.02	10.85^{f} ± 0.04	4.92^{g} ± 0.06	11.5^{f} ± 0.11	6.54 ^{h}± 0.08	3.25^{d} ± 0.37	9.77^{i} ± 0.20
20	EAH II	4.26^{a} ± 0.09	10.71^{b} ± 0.14	15.33^{c} ± 0.30	3.1^{d} ± 0.07	9.71^{e} ± 0.10	3.69^{d} ± 0.04	13.36^{f} ± 0.57	14.35^{f} ± 0.09	1.71^{g} ± 0.11	12.23^{h} ± 0.28
21	SAB I	34.51^{a} ± 0.86	31.75^{a} ± 1.10	11.98^{b} ± 0.12	14.21^{c} ± 0.05	17.67 ^{d}± 0.28	10.13^{e} ± 0.07	13.16 ^{f}± 0.38	19.99 ^{g}± 0.42	11.57^{b} ± 0.92	8.98^{h} ± 0.23
22	LA I	0.41^{a} ± 0.02	1.34^{b} ± 0.16	4.62^{c} ± 0.15	2.52^{d} ± 0.15	0.27^{e} ± 0.04	0.76^{f} ± 0.11	0.52^{a} ± 0.07	1.36 ^{b}± 0.01	6.1^{g} ± 0.61	20.31^{h} ± 1.10
23	RA	359.39^{a} ± 0.79	607.19^{b} ± 3.20	541.34^{c} ± 2.41	254.92^{d} ± 3.73	537.73^{e} ± 3.99	233.57 ^{f}± 3.51	386.01^{g} ± 0.09	346.64^{h} ±1.82	588.83^{d} ± 35.4	194.63^{i} ± 2.20
24	RAD	0.087^{a}± 0.01	0.12^{b} ± 0.01	0.3 ^{c}± 0.06	0.047^{cd} ± 0.03	0.01± 0.001	0.03 ± 0.02	0.047^{d} ± 0.01	0.03^{c} ± 0.01	0.13 ^{b}± 0.04	0.45^{d} ± 0.06
25	SAB II	15.94^{ae}± 1.12	30.3^{b} ± 1.59	40.77^{c} ± 1.01	11.74^{d} ± 1.03	14.07 ^{e}± 0.37	16.79^{f} ± 0.88	35.69^{g} ± 0.16	36.06 ^{g}± 0.48	4.48^{h} ± 1.30	0.87^{i} ± 0.03
26	CAA I	0.53^{a} ± 0.09	3.87^{b} ± 0.04	1.77^{c} ± 0.05	2.38^{d} ± 0.07	1.18^{e} ± 0.04	1.41^{f} ± 0.02	5.16^{g} ± 0.13	4.31 ^{b}± 0.16	0.55 ^{a}± 0.09	0.16^{h} ± 0.01
27	CAA II	2.36^{a} ± 0.11	3.91^{b} ± 0.09	1.41^{c} ± 0.09	2.39^{a} ± 0.09	3.13 ^{d}± 0.05	1.50 ^{c}± 0.02	1.84^{e} ± 0.07	2.23^{a} ± 0.06	0.00	0.45 ^{f}± 0.06
28	SAB III	132.46^{a} ± 0.82	203.67^{bg} ± 4.86	192.3^{b} ± 1.68	81.47^{c}± 1.68	110.64^{d} ± 0.42	75.93^{e} ± 0.29	116.2^{f} ± 1.75	206.33^{g} ± 2.17	48.06^{h} ± 14.48	8.61^{i}± 0.19
29	MR	4.66 ^{a}± 0.11	5.38^{b} ± 0.11	5.63^{b} ± 0.06	2.6^{c} ± 0.09	3.08 ^{d}± 0.02	2.16 ^{e}± 0.06	2.33^{e} ± 0.05	2.79^{c} ± 0.08	1.65^{f} ± 0.35	1.51^{f} ± 0.01
30	p-CAIE	0.15^{a}± 0.02	0.39^{b} ± 0.03	0.19^{a} ± 0.01	0.07 ^{c}± 0.007	0.12^{a} ± 0.06	0.02^{d} ± 0.01	0.09^{e} ± 0.01	0.16^{ad} ± 0.01	0.097^{e} ± 0.02	0.33^{b} ± 0.01
31	N B1	1.56^{a} ± 0.01	1.29 ^{b}± 0.10	1.33^{b} ± 0.01	1.55^{a} ± 0.041	1.67^{a} ± 0.05	1.08^{c} ± 0.03	1.24^{d} ± 0.04	1.6^{a} ± 0.07	3.29^{e} ± 1.28	2.04^{e} ± 0.04
32	NB2	9.25^{a} ± 0.04	7.68^{b} ± 0.26	3.48^{c} ± 0.01	1.53^{d} ± 0.047	3.11^{e} ± 0.05	2.11^{f} ± 0.07	2.89^{g} ± 0.09	1.45^{d} ± 0.06	4.83 ± 0.69	17.91^{h} ± 0.52

The results are means from 3 samples±standard error (SE).Means with the same letter with in each line are not statisticallyd ifferent in the Kruskal－Wallis test at $p \leqslant 0.05$

BTA-Benzoyl tartaric acid, SA- Syringic acid, EA- Epideoxyloganic acid, DAH-Dihydroxybenzoic acid hexoside, SAH- Salicylic acid-O-hexoside, CAH I-Caffeic acid hexoside I, , FA-Ferulic acid, CAH II -Caffeic acid hexoside II, SAGa- Sinapic acid-O-galactoside, PA -Prolithospermic acid, CA-Caffeic acid, SAGu-Sinapic acid-O-glucoside, PhA-Phaselic acid (2-O-caffeoylmalic acid), EC- Ethyl caffeate, PA I- Prolithospermic acid isomer, PA II- Prolithospermic acid isomer, TGG- tricaffeoyl-glucosyl-glucoside, SgA- Sagerinic acid, EAH I- Epideoxyloganic acid hexoside I, EAH II-Epideoxyloganic acid hexoside II, SAB I-Salvianolic acid B isomer I, LAI-Lithospermic acid isomer, RA-Rosmarinic acid, RAD-Rosmarinic acid derivative, SAB II-Salvianolic acid B isomer II, CAA I-Clinopodic acid A isomer I, CAA II-Clinopodic acid A isomer II, SAB III-Salvianolic acid B isomer III, MR-Methyl rosmarinate, pCAIE- p-Coumaric acid isoprenyl ester, NB1-Nepetoidin B1, NB2-Nepetoidin B2
