# Supplementary material for: Establishment of Shoot Cultures of Nepeta curviflora Boiss., Scale-Up in a Nutrient Sprinkle Bioreactor and Phytochemical Analysis
Source: Int J Mol Sci. 2025 Nov 25;26(23):11409. doi: 10.3390/ijms262311409 (PMC12692223; doi:10.3390/ijms262311409)
Supplement: Supplementary file 1 [file ijms-26-11409-s001.zip › Table S2.pdf]

Table S2. Productivity of total phenolic compounds in *in vitro* shoot cultures of *N. curviflora* cultured for 4 weeks in glass tubes (MS medium with 0.1 mg/L IAA and BAP or r-BAP at different concentrations) or in a nutrient sprinkle bioreactor in liquid MS medium with 0.1 mg/L IAA and 1.5 mg/L r-BAP.

***In vitro* plant material**	**Cytokinin concentration (mg/L)**	**Productivity (mg/L/day) ± SE**
Glass tubes MS + IAA + r-BAP	0.5	0.369 ± 0.0006
	**1.0**	**0.862 ± 0.006**
	**1.5**	**0.276 ± 0.001**
	2.0	0.327 ± 0.004
Glass tubes MS + IAA + BAP	0.5	0.529± 0.002
	1.0	0.204 ± 0.003
	1.5	0.476 ± 0.0009
	2.0	0.498 ± 0.002
Bioreactor MS + IAA + r-BAP	**1.5**	**2.256 ± 0.105**
