# Supplementary figures and images for: Establishment of Shoot Cultures of Nepeta curviflora Boiss., Scale-Up in a Nutrient Sprinkle Bioreactor and Phytochemical Analysis
Source: Int J Mol Sci. 2025 Nov 25;26(23):11409. doi: 10.3390/ijms262311409 (PMC12692223; doi:10.3390/ijms262311409)

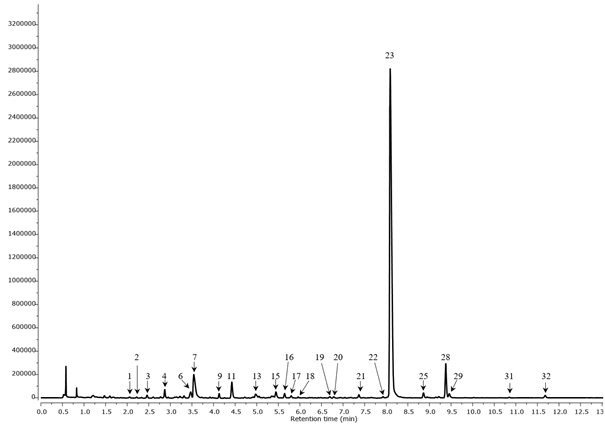

Supplement: Supplementary file 1 [file ijms-26-11409-s001.zip › Figure S1 chromatogram.tiff]
